# CO-DESIGNING RESEARCH IN COLLABORATION WITH PEOPLE WHO ARE RESIDENTS AND STAFF IN LONG-TERM CARE FOR OLDER ADULTS

**DOI:** 10.1093/geroni/igae098.3181

**Published:** 2024-12-31

**Authors:** Kay Shannon, David Jones, Katie Featherstone

**Affiliations:** Auckland University Of Technology, Auckland, Auckland, New Zealand; Manukau Institute of Technology, Ōtara, Auckland, New Zealand; University of West London, London, England, United Kingdom

## Abstract

One New Zealand provider of long-term care for older adults has shown fortitude in overcoming obstacles to developing a homelike facility where 81 residents live in small houses and continue with activities that connect them with lifelong identities. The CARE Village, Te Manaaki a Tura is the host organization for the sequential mixed methods study, which aims to compare resident experiences and outcomes in homelike and traditional facilities. Phase one determined the impact of the homelike model of care on International Resident Assessment Instrument (interRAI) quality indicators. The phase two qualitative study to understand the operationalization of nursing care at both types of facilities was co-designed with residents and staff of The CARE Village, Te Manaaki a Tura, including the staff-led Māori Cultural Awareness Team. The presentation reports on the co-design process. This presentation will discuss the co-design process and the meaningful contributions and insights to designing the qualitative research including developing questions for research participants and developing the strategy for analysing the data. Co-designers who are residents demonstrated their fortitude in continuing to provide service to the community. Co-design is becoming increasingly important as researchers and practitioners must include the perspectives of clients and other stakeholders in their work. Therefore, this presentation offers advice for those wanting to learn about the practical elements of the process.

